# An Analytical Mechanics Model for the Rotary Sliding Triboelectric Nanogenerator

**DOI:** 10.3390/mi15030371

**Published:** 2024-03-09

**Authors:** Guangping Gong, Maoyi Zhang, Dongqi An, Rui Li, Yewang Su

**Affiliations:** 1 State Key Laboratory of Structural Analysis, Optimization and CAE Software for Industrial Equipment, and International Research Center for Computational Mechanics, School of Mechanics and Aerospace Engineering, Dalian University of Technology, Dalian 116024, China; 2 State Key Laboratory of Nonlinear Mechanics, Institute of Mechanics, Chinese Academy of Sciences, Beijing 100190, China; 3 School of Engineering Science, University of Chinese Academy of Sciences, Beijing 100049, China

**Keywords:** triboelectric nanogenerator, elasticity, axisymmetric problem, stress function

## Abstract

In recent years, global attention towards new energy has surged due to increasing energy demand and environmental concerns. Researchers have intensified their focus on new energy, leading to advancements in technologies like triboelectrification, which harnesses energy from the environment. The invention of the triboelectric nanogenerator (TENG) has led to new possibilities, with the rotary sliding TENG standing out for its superior performance. However, understanding its mechanical behavior remains a challenge, potentially leading to structural issues. This paper introduces a novel analytical mechanics model to analyze the mechanical performance of the stator of the rotary sliding TENG, offering a new analytical solution. The solution also presents an innovative approach to solving axisymmetric problems in elasticity theory since it challenges a traditional assumption that the stress function depends solely on the radial coordinate, proposing a new stress function to derive a more general solution, supplementing the classical approach in the theory of elasticity. Through the obtained solutions, the mechanical characteristics of the rotary sliding TENG during operation are analyzed. A clearer relationship between mechanical characteristics and electrical output is expected to provide a theoretical basis for the design of the rotary sliding TENG.

## 1. Introduction

In recent years, global attention to the field of new energy has been increasing rapidly [1]. With the continuous growth of global energy demand and the rising awareness of environmental protection, new energy has become one of the important directions for global energy development. The development of new energy technologies not only helps to reduce reliance on traditional fossil fuels but also effectively reduces greenhouse gas emissions, providing new opportunities for global climate change mitigation. The application of clean energy, such as solar energy [2], wind energy [3], and geothermal energy [4], continues to expand, becoming an important choice for energy transformation. Within this framework, researchers have increased their investment in the field of new energy, accelerating the research, development, and application of new energy technologies, among which triboelectrification technology [5] has attracted considerable attention. The phenomenon of triboelectrification has piqued people’s interest over the last 2600 years [6]. With in-depth research into its mechanisms, this theory has gradually been translated into practical devices, successfully achieving energy harvesting from the living environment [7,8,9,10,11,12]. The invention of triboelectric nanogenerators (TENGs) is based on this enhanced understanding of the underlying mechanisms.

There are many types of TENGs, among which the more typical one is the rotary sliding TENG [13,14,15,16,17,18,19,20,21]. The rotary sliding TENG is an exceptional design within TENG structures, offering superior performances and greater adaptability to different forms of mechanical agitations. In recent years, many scholars have studied its electrical output characteristics. Jiang et al. [13] presented a theoretical model of the rotary sliding disk TENG, which includes dielectric-to-dielectric and conductor-to-dielectric cases working in both contact and non-contact modes. It was found that both the triboelectric material and structural parameters are relevant to the basic properties of the disk TENG, such as short-circuit transferred charges, open-circuit voltage, and capacitance through finite element analysis. By numerically calculating the approximate semi-analytical equations of output voltage, the amount of charge transferred between electrodes, and the rotation angle, the resistive load output characteristics of the rotary sliding disk TENG were investigated. The influences of structural parameters and operating conditions on the overall performance of the device were discussed, which were useful for guiding the design of the device structure. Bai et al. [14] implemented a charge pumping technique using an innovative synchronous rotation design, facilitating the injection of bound charges from the pumping TENG into the primary TENG. This approach effectively increased the charge density. In comparison to traditional TENGs, the charge density saw a remarkable ninefold increase, while the average power surged by over 15 times. This method effectively addressed the power output constraints in mechanical energy harvesting, laying the foundation for high-power self-sustaining systems and extensive environmental energy harvesting initiatives. Xie et al. [15] developed a TENG based on the traditional vertical wind-cup structure, effectively harvesting wind energy. This rotary TENG produced an open-circuit voltage of 250 V and a short-circuit current of 0.25 mA, reaching a peak power output of 62.5 mW at a wind speed of 15 m/s. This innovative integration of TENGs with conventional wind power technology marked a significant advancement with promising prospects. Rodrigues et al. [16] developed a TENG using polytetrafluoroethylene and Nylon 6.6 as triboelectric materials, effectively harvesting energy from water flows. This rotary TENG produced an average voltage value of 102.2 V, a short-circuit current density of 120 mA/m^2^, and a maximum power density of 6.1 W/m^2^. To harvest linear mechanical energy, Tcho et al. [17] introduced an innovative disk-based TENG that, when paired with a gear system, effectively transformed linear mechanical energy into rotational energy and ultimately into electrical energy. Experimental findings demonstrated that, under identical conditions, the electrical output of this new TENG surpassed that of the TENG utilizing the vertical contact separation mode. Zhong et al. [18] presented an easily assembled electromagnetic-triboelectric hybrid nanogenerator driven by magnetic coupling to harvest fluid energy. Through the magnetic coupling, the encapsulation, installation and maintenance of the hybrid nanogenerator are much easier, which makes the nanogenerator more stable.

However, in the actual operation of the generator, due to friction between two contacting triboelectric layers, the output power and life of the generator are affected. In order to reduce this friction, many scholars have conducted research and improved the design. Du et al. [19] introduced a pioneering ferromagnetic metal particle-based TENG, which utilized the infinite point contact of rolling motion instead of the planar contact of film material, thereby reducing abrasion. Additionally, the extensive increase in the contact area of the ferromagnetic metal particles enhanced the electrical output of the ferromagnetic metal particle-based TENG, achieving a charge density of 103 μC/m^2^ and a peak power density of 400 mW/m^2^/Hz. During the experiment, after an initial running-in period of 10,000 cycles, the ferromagnetic metal particle-based TENG exhibited remarkable durability, maintaining 97% of the output charge over 110,000 cycles. This study offered a dependable method for enhancing the durability of sliding TENG. Ramaswamy et al. [20] incorporated diamond-like carbon (DLC) film, a highly efficient triboelectric material, into the TENG, significantly enhancing its durability. The DLC films were applied to both the substrate and the electrode of the contact-separation TENG using a plasma-based ion implantation and deposition technique, effectively reducing their friction coefficient. During the durability assessment, the DLC-coated rotary sliding TENG maintained a consistent output current for 3 h. In addition, DLC films are more advantageous due to their thinness and more controllable thickness compared to other dielectric materials. Zhang et al. [21] introduced a new surface-textured film with self-lubricating properties to the TENG as a tribo-material. This resulted in a significant reduction in both the static and dynamic coefficients of friction of the material, from 1.802 to 0.209 and from 1.403 to 0.195, respectively, thereby greatly enhancing its mechanical durability. In the experiment, the textured film and self-adapting contact-synergized bidirectional TENG obtained demonstrated remarkable output stability and outstanding mechanical durability over 350,000 cycles. This novel and straightforward method effectively improved the durability of the sliding freestanding TENG. Hao [22] developed an innovative coaxial rolling charge pump TENG that harnessed wind energy. The rolling friction charge pump TENG transferred positive and negative charges directly to the main TENG, offering higher durability compared to sliding friction. This approach also significantly enhanced charge density and output power at the same time. Experimental findings demonstrated that the output voltage of the coaxial rolling charge pump TENG could increase by 5800% with the charge pump supplementary charging strategy. Moreover, the coaxial rolling charge pump TENG maintained stable output performance even after 72,000 cycles. This study underscored the significant potential of utilizing environmental energy sources to power smart IoT nodes. In conclusion, most studies have focused on reducing the friction by applying new materials that have lower coefficients of friction or transferring sliding friction mode to rolling friction mode. However, the current understanding of the mechanical performance of TENGs is still insufficient, which may lead to some issues such as premature structure failure [23]. Therefore, in-depth research into their mechanical performance is particularly important. The core of the rotary sliding TENG consists of two parts, namely the stator and the rotator. The rotator rotates, coming into contact and friction with the stator, causing a transfer of electric charge between the electrodes, thereby achieving the conversion of mechanical energy into electrical energy and thus the effect of power generation.

This paper develops a novel analytical mechanics model, which is used to analyze the mechanical performance of the stator of the rotary sliding TENG. The model is extensively discussed in Section 2, with the corresponding general equations provided. The new analytical solution to the problem is deduced and discussed in Section 3. The conclusions are described in Section 4.

## 2. Analytical Mechanics Model of the Rotary Sliding TENG

**Figure 1:**
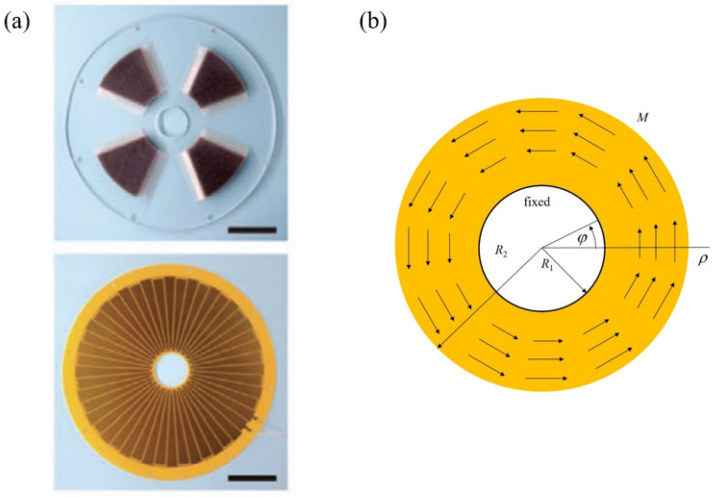
(**a**) Photographs of the stators of the rotary sliding TENG. Scale bar: 5 cm. Adapted with permission from [14]. Copyright 2020, John Wiley and Sons. (**b**) Schematic diagram of the model for the rotary sliding TENG corresponding to (**a**). Arrows indicate the moment *M*.

The stator and rotator are subjected to an equal but opposite external load. Compared to the stator, the rotator is subjected to inertial force due to high-speed rotation such that this factor needs to be taken into account when analyzing the rotator, making the analysis much more complicated. Therefore, in this paper, we focus on the stator. Figure 1a presents the photographs of the stators of the rotary sliding TENG, and Figure 1b illustrates a schematic diagram of the corresponding model, where the yellow area represents a ring-shaped sliding disk with the inner side fixed and the outer side free, subjected to a torsional moment. The dimensions of the ring are such that the inner radius is $R_{1}$ and the outer radius is $R_{2}$. The entire ring is subjected to a torsional moment of magnitude M. A polar coordinate system is established, with the origin located at the center of the ring.

In the polar coordinate system, the equations of equilibrium, geometric relations, and Hooke’s law for the plane stress problems are as follows [24].

(1) $$\begin{array}{l} \frac{\partial \sigma_{\rho }}{\partial \rho }+\frac{1}{\rho }\frac{\partial \tau_{\rho \varphi }}{\partial \varphi }+\frac{\sigma_{\rho }-\sigma_{\varphi }}{\rho }+f_{\rho }=0,\\ \frac{1}{\rho }\frac{\partial \sigma_{\varphi }}{\partial \varphi }+\frac{\partial \tau_{\rho \varphi }}{\partial \rho }+\frac{2\tau_{\rho \varphi }}{\rho }+f_{\varphi }=0, \end{array}$$

(2) $$\begin{array}{l} \varepsilon_{\rho }=\frac{\partial u_{\rho }}{\partial \rho },\\ \varepsilon_{\varphi }=\frac{u_{\rho }}{\rho }+\frac{1}{\rho }\frac{\partial u_{\varphi }}{\partial \varphi },\\ \gamma_{\rho \varphi }=\frac{1}{\rho }\frac{\partial u_{\rho }}{\partial \varphi }+\frac{\partial u_{\varphi }}{\partial \rho }-\frac{u_{\varphi }}{\rho }, \end{array}$$

(3) $$\begin{array}{l} \varepsilon_{\rho }=\frac{1}{E}\left(\sigma_{\rho }-\nu \sigma_{\varphi }\right),\\ \varepsilon_{\varphi }=\frac{1}{E}\left(\sigma_{\varphi }-\nu \sigma_{\rho }\right),\\ \gamma_{\rho \varphi }=\frac{2\left(1+\nu \right)}{E}\tau_{\rho \varphi }, \end{array}$$

where $\sigma_{\rho }$ and $\sigma_{\varphi }$ are the radial and circumferential normal stresses, $\tau_{\rho \varphi }$ is the shearing stress, $\varepsilon_{\rho }$, $\varepsilon_{\varphi }$, and $\gamma_{\rho \varphi }$ are the strain components, $u_{\rho }$ and $u_{\varphi }$ are the displacement components, $f_{\rho }$ and $f_{\varphi }$ are the components of the body force per unit volume, ν is Poisson’s ratio, and E is Young’s modulus. 

When the body force vanishes, the compatibility equation is

(4) $${\left(\frac{\partial^{2}}{\partial \rho^{2}}+\frac{1}{\rho }\frac{\partial }{\partial \rho }+\frac{1}{\rho^{2}}\frac{\partial^{2}}{\partial \varphi^{2}}\right)}^{2}\Phi =0,$$

where Φ is the stress function. The stress components in terms of Φ are represented as

(5) $$\begin{array}{l} \sigma_{\rho }=\frac{1}{\rho }\frac{\partial \Phi }{\partial \rho }+\frac{1}{\rho^{2}}\frac{\partial^{2}\Phi }{\partial \varphi^{2}},\\ \sigma_{\varphi }=\frac{\partial^{2}\Phi }{\partial \rho^{2}},\\ \tau_{\rho \varphi }=-\frac{\partial }{\partial \rho }\left(\frac{1}{\rho }\frac{\partial \Phi }{\partial \varphi }\right). \end{array}$$

## 3. Solution and Results of the Model

In elasticity, the stress function approach is often adopted to solve a two-dimensional problem. For an axisymmetric problem, the classical solution procedure starts with the prior hypothesis that the stress function is independent of the circumferential variable. In fact, the classical solution is narrowly applicable to the problems with zero circumferential quantities, i.e., circumferential shearing stress, shearing strain, and displacement. These quantities, however, do not have to be zero in a general axisymmetric problem, where the independence of φ for all the physical quantities still holds. In this section, based on the characteristics of axisymmetric problems, we start with the analysis of the axisymmetric stresses, which have intuitional physical meanings in comparison with the stress function.

First, $\tau_{\rho \varphi }$ depends only on ρ in the axisymmetric problem; thus, from the third equation of Equation (5), we obtain the following equations:

(6) $$\tau_{\rho \varphi }=-\frac{\partial }{\partial \rho }\left(\frac{1}{\rho }\frac{\partial \Phi }{\partial \varphi }\right)=f_{1}\left(\rho \right),$$

where $f_{1}\left(\rho \right)$ is a function of ρ only. By integration, we have

(7) $$\frac{1}{\rho }\frac{\partial \Phi }{\partial \varphi }=-\int f_{1}\left(\rho \right)d\rho +f_{2}\left(\varphi \right),$$

where $f_{2}\left(\varphi \right)$ is a function of φ only. Further integration gives the following:

(8) $$\Phi =-\varphi \rho \int f_{1}\left(\rho \right)d\rho +\rho \int f_{2}\left(\varphi \right)d\varphi +f_{3}\left(\rho \right),$$

with $f_{3}\left(\rho \right)$ as a function of ρ only. Equation (8) is rewritten as

(9) $$\Phi =\Phi_{1}\left(\rho \right)+\varphi \Phi_{2}\left(\rho \right)+\rho \Phi_{3}\left(\varphi \right),$$

where $\Phi_{1}\left(\rho \right)=f_{3}\left(\rho \right)$, $\Phi_{2}\left(\rho \right)=-\rho \int f_{1}\left(\rho \right)d\rho$, and $\Phi_{3}\left(\varphi \right)=\int f_{2}\left(\varphi \right)d\varphi$. Substituting Equation (9) into the second equation of Equation (5) gives the following:

(10) $$\sigma_{\varphi }=\frac{d^{2}\Phi_{1}\left(\rho \right)}{d\rho^{2}}+\varphi \frac{d^{2}\Phi_{2}\left(\rho \right)}{d\rho^{2}}.$$

Since $\sigma_{\varphi }$ depends only on ρ, we obtain the following equation:

(11) $$\frac{d^{2}\Phi_{2}\left(\rho \right)}{d\rho^{2}}=0$$

such that

(12) $$\Phi_{2}\left(\rho \right)=C_{0}\rho +C_{1},$$

where $C_{0}$ and $C_{1}$ are the integration constants. Substituting Equation (9) into the first equation of Equation (5) gives the following:

(13) $$\begin{array}{cc} \sigma_{\rho } & =\frac{1}{\rho }\frac{\partial \Phi }{\partial \rho }+\frac{1}{\rho^{2}}\frac{\partial^{2}\Phi }{\partial \varphi^{2}}\\ & =\frac{1}{\rho }\frac{d\Phi_{1}\left(\rho \right)}{d\rho }+\frac{1}{\rho }\left[\frac{d^{2}\Phi_{3}\left(\varphi \right)}{d\varphi^{2}}+\Phi_{3}\left(\varphi \right)+\varphi \frac{d\Phi_{2}\left(\rho \right)}{d\rho }\right]. \end{array}$$

Since $\sigma_{\rho }$ depends only on ρ, by taking into account Equation (12), we obtain the following:

(14) $$\frac{d^{2}\Phi_{3}\left(\varphi \right)}{d\varphi^{2}}+\Phi_{3}\left(\varphi \right)+C_{0}\varphi =C_{2}$$

such that

(15) $$\Phi_{3}\left(\varphi \right)=C_{3}\sin\varphi +C_{4}\cos\varphi -C_{0}\varphi +C_{2},$$

where $C_{2}$, $C_{3}$, and $C_{4}$ are the constants to be determined. Substituting Equations (12) and (15) into Equation (9) gives the following equation:

(16) $$\Phi =\Phi_{1}\left(\rho \right)+C_{1}\varphi +C_{2}\rho +C_{3}\rho \sin\varphi +C_{4}\rho \cos\varphi .$$

$C_{2}\rho$ can be incorporated into $\Phi_{1}\left(\rho \right)$; thus, we can write the following equation as:

(17) $$\Phi =\Phi_{1}\left(\rho \right)+C_{1}\varphi +C_{3}\rho \sin\varphi +C_{4}\rho \cos\varphi .$$

The compatibility equation must be satisfied by substituting Equation (17) into Equation (4), which yields the following:

(18) $${\left(\frac{d^{2}}{d\rho^{2}}+\frac{1}{\rho }\frac{d}{d\rho }\right)}^{2}\Phi_{1}\left(\rho \right)=0.$$

It is noted that $C_{1}\varphi$, $C_{3}\rho \sin\varphi$, and $C_{4}\rho \cos\varphi$ on the right-hand side of Equation (17) automatically satisfy Equation (4). Therefore, Equation (17) is the stress function for a two-dimensional axisymmetric problem, and $\Phi_{1}\left(\rho \right)$ is the stress function obtained in the previous study [24] by assuming the dependence on the radial coordinate only. 

The ordinary differential Equation (18) has the following general solution [24]:

(19) $$\Phi_{1}\left(\rho \right)=A\ln\rho +B\rho^{2}\ln\rho +C\rho^{2}+D,$$

where A, B, C, and D are the constants. Substituting Equation (17) into Equation (5), the stresses in terms of the stress function are as follows:

(20) $$\sigma_{\rho }=\frac{1}{\rho }\frac{d\Phi_{1}\left(\rho \right)}{d\rho },\;\sigma_{\varphi }=\frac{d^{2}\Phi_{1}\left(\rho \right)}{d\rho^{2}},\;\tau_{\rho \varphi }=\frac{C_{1}}{\rho^{2}}.$$

It is seen from Equation (20) that the terms $C_{3}\rho \sin\varphi$ and $C_{4}\rho \cos\varphi$ in Equation (17) do not contribute to the stresses and are thus ignored, such that the stress function becomes the following:

(21) $$\Phi =\Phi_{1}\left(\rho \right)+C_{1}\varphi =A\ln\rho +B\rho^{2}\ln\rho +C\rho^{2}+D+C_{1}\varphi .$$

Compared to the traditional solution [24], it is interesting that there is a supplementary term $C_{1}\varphi$ in the present solution.

By substituting Equation (21) into Equation (5) and then, into Equation (3), the stress and strain are expressed as:

(22) $$\begin{array}{l} \sigma_{\rho }=\frac{A}{\rho^{2}}+B\left(1+2\ln\rho \right)+2C,\\ \sigma_{\varphi }=-\frac{A}{\rho^{2}}+B\left(3+2\ln\rho \right)+2C,\\ \tau_{\rho \varphi }=\frac{C_{1}}{\rho^{2}}, \end{array}$$

and

(23) $$\begin{array}{l} \varepsilon_{\rho }=\frac{1}{E}\left[\left(1+\nu \right)\frac{A}{\rho^{2}}+\left(1-3\nu \right)B+2\left(1-\nu \right)B\ln\rho +2\left(1-\nu \right)C\right],\\ \varepsilon_{\varphi }=\frac{1}{E}\left[-\left(1+\nu \right)\frac{A}{\rho^{2}}+\left(3-\nu \right)B+2\left(1-\nu \right)B\ln\rho +2\left(1-\nu \right)C\right],\\ \gamma_{\rho \varphi }=\frac{2\left(1+\nu \right)}{E}\frac{C_{1}}{\rho^{2}}. \end{array}$$

By substituting Equations (22) and (23) into the first and second equations of Equation (2), we obtain the following:

(24) $$\begin{array}{cc} u_{\rho } & =\frac{1}{E}\left[-\frac{\left(1+\nu \right)A}{\rho }+\left(1-3\nu \right)B\rho +2\left(1-\nu \right)B\rho \left(\ln\rho -1\right)+2\left(1-\nu \right)C\rho \right]\\ & +g_{1}\left(\varphi \right),\\ u_{\varphi } & =\frac{4B\rho \varphi }{E}-\int g_{1}\left(\varphi \right)d\varphi +g_{2}\left(\rho \right). \end{array}$$

where $g_{1}\left(\varphi \right)$ and $g_{2}\left(\rho \right)$ are functions of φ and ρ, respectively, to be determined later. By substituting Equation (24) into the third equation of Equation (2), we obtain the following:

(25) $$\frac{dg_{1}\left(\varphi \right)}{d\varphi }+\int g_{1}\left(\varphi \right)d\varphi =g_{2}\left(\rho \right)-\rho \frac{dg_{2}\left(\rho \right)}{d\rho }+\frac{2\left(1+\nu \right)}{E}\frac{C_{1}}{\rho }.$$

Equation (25) holds only if both sides are equal to the same constant *F*, i.e.,

(26) $$\begin{array}{l} \frac{dg_{1}\left(\varphi \right)}{d\varphi }+\int g_{1}\left(\varphi \right)d\varphi =F,\\ g_{2}\left(\rho \right)-\rho \frac{dg_{2}\left(\rho \right)}{d\rho }+\frac{2\left(1+\nu \right)}{E}\frac{C_{1}}{\rho }=F. \end{array}$$

The first equation of Equation (26) gives the following:

(27) $$\begin{array}{l} g_{1}\left(\varphi \right)=I\cos\varphi +K\sin\varphi ,\\ \int g_{1}\left(\varphi \right)d\varphi =F+I\sin\varphi -K\cos\varphi , \end{array}$$

and the second equation of Equation (26) gives the following equation:

(28) $$g_{2}\left(\rho \right)=H\rho +F-\frac{1+\nu }{E}\frac{C_{1}}{\rho },$$

where *I*, *K*, and *H* are the integration constants. By substituting Equations (27) and (28) into Equation (24), we have the displacement solution:

(29) $$\begin{array}{cc} u_{\rho } & =\frac{1}{E}\left[-\left(1+\nu \right)\frac{A}{\rho }+\left(1-3\nu \right)B\rho +2\left(1-\nu \right)B\rho \left(\ln\rho -1\right)+2\left(1-\nu \right)C\rho \right]\\ & +I\cos\varphi +K\sin\varphi ,\\ u_{\varphi } & =\frac{4B\rho \varphi }{E}-I\sin\varphi +K\cos\varphi +H\rho -\frac{\left(1+\nu \right)}{E}\frac{C_{1}}{\rho }, \end{array}$$

The boundary conditions are as follows:

(30) $$\begin{array}{l} {\left.u_{\rho }\right|}_{\rho =R_{1}}=0,\;{\left.\;u_{\varphi }\right|}_{\rho =R_{1}}=0,\int_{R_{1}}^{R_{2}}\tau_{\rho \varphi }\rho d\rho =M,\\ {\left.\sigma_{\rho }\right|}_{\rho =R_{2}}=0,\;{\left.\sigma_{\varphi }\right|}_{\rho =R_{2}}=0,{\left.u_{\varphi }\right|}_{\varphi =\varphi_{0}}={\left.u_{\varphi }\right|}_{\varphi =\varphi_{0}+2\pi }, \end{array}$$

and we obtain the following:

(31) $$\begin{array}{l} A=0,\;B=0,\;C=0,\;I=0,\;K=0,\\ C_{1}=\frac{M}{\ln R_{2}\;-\;\ln R_{1}},H=\frac{M\left(1\;+\;\nu \right)}{R_{1}^{2}E\left(\ln R_{2}\;-\;\ln R_{1}\right)}. \end{array}$$

Therefore, the displacement solutions are as follows:

(32) $$\begin{array}{l} u_{\rho }=0,\\ u_{\varphi }=\frac{M\left(1\;+\;\nu \right)}{E\left(\ln R_{2}\;-\;\ln R_{1}\right)}\left(\frac{\rho }{R_{1}^{2}}-\frac{1}{\rho }\right), \end{array}$$

the stress solutions are as follows:

(33) $$\begin{array}{l} \sigma_{\rho }=0,\\ \sigma_{\varphi }=0,\\ \tau_{\rho \varphi }=\frac{M}{\rho^{2}\left(\ln R_{2}\;-\;\ln R_{1}\right)}, \end{array}$$

and the strain solutions are as follows:

(34) $$\begin{array}{l} \varepsilon_{\rho }=0,\\ \varepsilon_{\varphi }=0,\\ \gamma_{\rho \varphi }=\frac{2\left(1\;+\;\nu \right)}{E}\frac{M}{\rho^{2}\left(\ln R_{2}\;-\;\ln R_{1}\right)}. \end{array}$$

From the displacement solution, it is evident that the primary form of displacement is circumferential, with no axial displacement. The derivative of the circumferential displacement is positive, which indicates that the circumferential displacement does not increase linearly with the radial coordinate and also suggests that the circumferential displacement on the outer side of the ring will increase rapidly with the radial coordinate. By maintaining the inner radius as a constant, increasing the outer radius will lead to a larger displacement all over the stator. 

From the stress solution, it is seen that the main form of stress is shear stress, with zero axial normal stress and circumferential stress. The shear stress is maximum on the inner side of the ring of magnitude $M/\left[R_{1}^{2}\left(\ln R_{2}-\ln R_{1}\right)\right]$. The outer side exhibits the minimum shear stress but maintains a non-zero value. 

From the strain solution, it is seen that the main form of strain is shear strain, which is linearly related to stress, with zero axial and circumferential normal strain. The maximum strain occurs on the inner side of the ring of magnitude $2M\left(1+\nu \right)/\left[ER_{1}^{2}\left(\ln R_{2}-\ln R_{1}\right)\right]$. The outer side exhibits the minimum shear strain but maintains a non-zero value.

**Figure 2:**
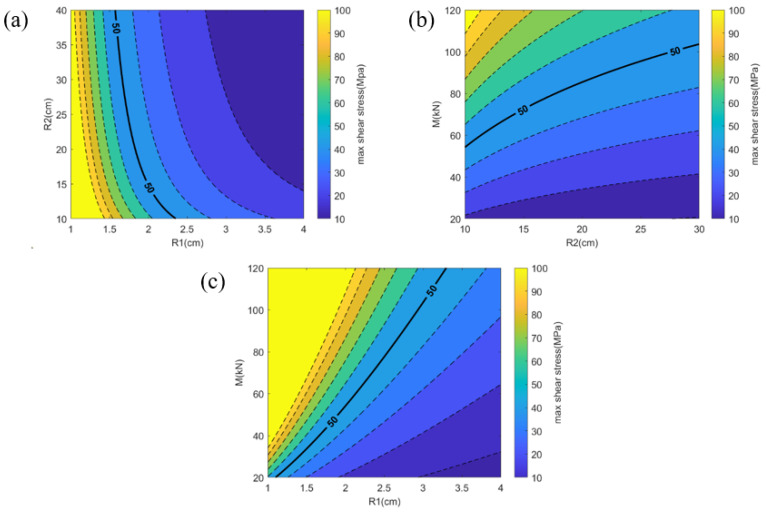
(**a**) Contour map of the maximum shear stress as a function of the inner and outer radii. (**b**) Contour map of the maximum shear stress as a function of the outer radius and moment. (**c**) Contour map of the maximum shear stress as a function of the inner radius and moment.

In order to provide more intuitive guidance for the design of the rotary sliding TENG, we plotted contour maps of the maximum shear stress as a function of inner and outer radii, of outer radius and moment, and of inner radius and moment, as shown in Figure 2a, Figure 2b, and Figure 2c, respectively, via the MATLAB software (MATLAB Version R2021b, MathWorks, USA). In these contour maps, the color changes from yellow to purple represent the transition from higher to lower maximum stress magnitude. The bold solid lines represent the allowable maximum shear stress. When the maximum shear stress exceeds the allowable stress, the design is considered unsafe, whereas when it is below the allowable stress, the design is deemed safe. It is important to note that different materials have different allowable stresses. In this study, the allowable stress is 50 MPa [25].

Specifically, Figure 2a describes the relationship between the maximum shear stress and the inner and outer radii. The horizontal axis represents the inner radius, ranging from 1 to 4 cm, while the vertical axis represents the outer radius, ranging from 10 to 30 cm. Additionally, the value of *M* is 40 kN in this study. The area to the right of the solid line in Figure 2a represents the safe zone. Figure 2b illustrates the relationship between the maximum shear stress and the outer radius and bending moment. The horizontal axis represents the outer radius, ranging from 10 to 30 cm, while the vertical axis represents the bending moment, ranging from 20 kN to 120 kN. The value of the inner radius is 3 cm in this study [14]. In Figure 2b, the safe zone is located below the solid line. Figure 2c illustrates the relationship between the maximum shear stress and the inner radius and bending moment. The horizontal axis represents the inner radius, ranging from 1 to 4 cm, while the vertical axis represents the bending moment, ranging from 20 kN to 120 kN. The value of the outer radius is 30 cm in this study [14]. In Figure 2c, the safe zone is located below the solid line.

The analytical solution obtained in this study reveals that the maximum shear stress is influenced by the inner and outer radii as well as the moment. The approximate range for the inner and outer radii can be inferred from prior research [14]. The moment is directly proportional to the frictional force; however, distinct TENGs are associated with varying frictional forces. From the previous study [19,20,21,22], it seems that when the friction force changes, the electrical output is different. However, this relationship is currently unclear, requiring further investigation. Once the connection between electrical output and friction force is established, the relationship between electrical output and mechanical performance will become clearer. 

The present new analytical mechanics model not only helps to better understand the mechanical characteristics of the rotary sliding TENG but also provides a new research perspective for the two-dimensional axisymmetric problems. This will help to further expand our understanding of other types of rotating generators, such as electromagnetic generators [26] and electrostatic current generators [27]. By applying the model to other types of generators, its potential applications in different engineering fields can be explored.

## 4. Conclusions

In this paper, a novel analytical mechanics model is introduced to examine the mechanical performance of the stator in the rotary sliding TENG, offering an innovative analytical solution. A new stress function-based solution is derived from the analysis of the stresses that are required to be axisymmetric according to the characteristics of axisymmetric problems. Through a strict derivation, a new solution is obtained, revealing that the classical solution has overlooked a significant term representing pure circumferential shear. The finding is expected to serve as an important supplement to the classical solution in the theory of elasticity. Contour maps illustrating the relationship between maximum shear stress and inner radius, outer radius, and moment have been created. Highlighting the allowable stress on the graph facilitates improved design by ensuring the selection of appropriate parameters to maintain the maximum stress within the allowable stress range, thus providing more intuitive guidance for the design of the rotary sliding TENG. The correlation between mechanical performance and electrical output requires additional investigation as it is anticipated to establish a foundation for the comprehensive design of TENGs.

## Data Availability

The data presented in this study are available on request from the corresponding author.
